# Analysis of Elevated Liver Enzymes in an Acute Medical Setting: Jaundice May Indicate Increased Survival in Elderly Patients with Bacterial Sepsis

**DOI:** 10.4103/1319-3767.70609

**Published:** 2010-10

**Authors:** Amir A. Shah, Michael Patton, Wajahat H. Chishty, Amir Hussain

**Affiliations:** Crosshouse Hospital, Kilmarnock KA2 0BE, Scotland, UK; 1 Department of Computing Science, University of Stirling, Stirling FK9 4LA, Scotland, UK

**Keywords:** Alcohol-related liver disease, elevated LFTs, septicemia in elderly

## Abstract

**Background/Aim:**

It has been shown previously that in primary care settings in UK abnormal liver enzymes are not adequately investigated and followed up; hence potentially treatable chronic liver diseases remain undiagnosed. No such published data is available with regard to secondary care settings. The aims of this audit were, to determine if the current practice in our hospital with regards to investigation, management and follow-up of patients with elevated liver enzymes is in accordance with American Gastroenterology Association (AGA) guidelines and to analyze the effect of age and elevated parameters of liver blood tests on mortality in patients with bacterial sepsis.

**Materials and Methods:**

A total of 4816 patients were admitted to our acute medical receiving unit during a period of 6 months, of which 378 were with elevated liver enzymes.

**Results and Conclusion:**

The common conditions that resulted in elevated liver enzymes were sepsis (123) and alcohol-related liver diseases (120). All patients with elevated parameters of liver function tests (LFTs) were fully investigated, managed and followed up in accordance with AGA guidelines. In addition, in patients with bacterial sepsis, old age was associated with increased mortality, while development of jaundice in elderly patients with bacterial sepsis was associated with increased survival.

It has been demonstrated previously that in primary care settings, cases with abnormal parameters of liver function tests (LFTs) are not adequately investigated and followed up; hence potentially treatable chronic liver diseases remain undiagnosed.[1] No such published data is available with regard to secondary care settings, where more serious patients are admitted for urgent investigation and treatment. Therefore, a retrospective audit was undertaken to study if the current practice in our busy district general hospital with regard to investigation, diagnosis, management and follow-up of patients with elevated liver enzymes is in accordance with American Gastroenterology Association (AGA) guidelines, which state that patients with abnormal LFT parameters should either be followed up until these parameters become normal or investigated in a stepwise manner until a cause is found.[2]

In addition, bacterial sepsis can cause liver damage by various mechanisms.[3–8] However, it is not clear if development of jaundice has any prognostic value in these patients. Furthermore, age is an important factor that determines the outcome of treatment in patients with sepsis. It has been demonstrated that elderly patients are more likely to succumb to infection because of low immunity at old age.[9,10] Therefore, we analyzed the effect of age and elevated parameters of liver blood tests on mortality in patients with bacterial sepsis admitted to our acute medical unit.

## MATERIALS AND METHODS

Retrospective analysis of patients with elevated parameters of liver blood tests who were admitted to our acute medical admission unit over a period of six months from 1^st^ July 2006 to 31^st^ December 2006 was conducted. Result of liver blood test was defined as elevated if any one of the following results was found: transaminases (AST and ALT) were >100 U/L, alkaline phosphatase was >250 μmol/L or bilirubin was >50 μmol/L.

These patients were identified from the computer records of the Biochemistry Department.

To analyze the impact of age and LFT parameters on mortality of patients with bacterial sepsis, these patients were divided into three groups on the basis of their age. Group 1 (*n*=33) included patients between the ages of 19 and 49 years; Group 2 (*n*=67), between 49 and 80 years; and group 3 (n=23), patients over 80 years. In each group, the effect of age and LFT parameters on mortality was analyzed separately. All comparisons were performed using Student *t* test for unpaired determinations. The criterion for statistical significance was *P* < 0.05. The audit was approved by the Clinical Effectiveness Department of the hospital.

## RESULTS

Out of 4816 patients admitted to our acute medical receiving unit during the period of 6 months, 378 (8%) had elevated liver enzymes, of whom 204 were males. The diagnosis of these patients is summarized in Table 1.

**Table 1:** Diagnosis in 378 patients with elevated liver function tests parameters

Diagnosis	Total
Bacterial sepsis	123
Alcohol-related elevated liver enzymes (without decompensation)	82
De-compensated chronic alcoholic liver disease	48
Tumors (primary and secondary)	39
Congestive cardiac failure	19
Drugs-related abnormal LFT parameters (statins, antibiotics, tegretal and azathioprine)	18
Drug overdose (paracetamol)	13
Hepatitis B and C	7
Nonalcoholic fatty liver disease	7
Autoimmune hepatitis	6
Pancreatitis	6
Miscellaneous (viral, primary biliary cirrhosis, primary sclerosing cholangitis, sphincter of oddi dysfunction, vasculitis and hemolysis)	10

Among 378 patients, 100 (26%) patients with elevated liver enzymes died within 30 days of admission — 42% died of sepsis, 27% died due to malignancy, 22% succumbed to various complications of chronic de-compensated liver disease, while remaining 9% died of various other causes, such as heart failure or pulmonary embolism.

In the cohort of 123 patients with sepsis, pneumonia (53) was the most common cause of elevated LFT parameters, followed by biliary tract infection (38) and urinary tract infections (29). Various other infections that resulted in elevated LFT parameters included 2 cases of hepatic abscesses and 1 case of staph aureous endocarditis.

Analysis of mortality in patients with sepsis showed that pneumonia was associated with the highest mortality (25/53), followed by urosepsis (8/29) and biliary tract infections (3/38); while 1 patient each with liver abscess and infective endocarditis died.

Among the 278 alive patients, 168 (58%) were being followed up in consultant-led clinics, while the remaining 42% were referred back to primary care after being fully investigated and adequately managed in the hospital, or were followed up in nurse-led clinics.

**Figure 1:**
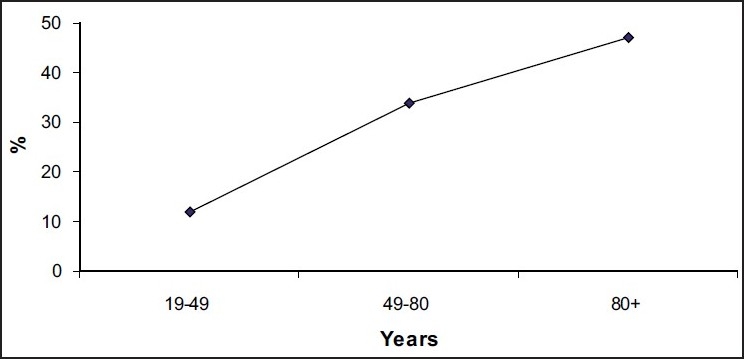
Effect of age on mortality due to sepsis

The effect of age and elevated LFT parameters on mortality in each group of patients with bacterial sepsis is shown in Figure 1 and Table 2, respectively.

**Table 2:** Effect of mean values of transaminases (ALT and AST) and bilirubin on mortality in patients with bacterial sepsis

		ALT (U/L)	*p* value	AST (U/L)	*p* value	Bilirubin (μmol/L)	*p* value
Group 1	Dead (*n*=4)	411	0.6	655	0.19	135	0.16
	Alive (*n*=29)	245		319		53	
Group 2	Dead (*n*=23)	152	0.20	290	0.49	80	0.32
	Alive (*n*=44)	360		598		54	
Group 3	Dead (*n*=11)	326	0.16	592	0.23	23	0.012*
	Alive (*n*=12)	109		113		93	

* *P* < 0.05 was considered significant

## DISCUSSION

Sherwood *et al*. published an audit on treatment in primary care setting, which demonstrated that patients with abnormal liver enzymes (in LFTs) were not adequately investigated and followed up and therefore potentially treatable chronic liver diseases remained undiagnosed.[1] However, no such data was available regarding treatment in secondary care settings, where more serious patients are managed. We therefore designed a retrospective study to evaluate if our current practice is in accordance with AGA guidelines, with regard to investigation, diagnosis, management and follow-up of patients with elevated liver enzymes. In addition, we also analyzed the impact of age and elevated LFT parameters on mortality of patients with bacterial sepsis.

We defined “elevated” liver blood test parameters as those that were at least twice the upper limit of the local reference range. These values were chosen because a small rise in liver blood test parameters can occur even in minor and nonsignificant ailments.

The type of patients analyzed in our study was different from that in Sherwood’s audit. Patients in our study were admitted to the acute medical receiving unit whereas patients in Sherwood’s audit were seen in primary care setting. Therefore, most of our patients were either suffering from life-threatening conditions such as septicemia, alcohol-related liver disease or other serious conditions that needed urgent investigations and management.

Substantial number of our patients had alcohol-related elevated liver biochemistry; 48 of these 130 patients had de-compensated liver disease and were frequently admitted to our unit with various complications. They were regularly being followed up in our hepatology clinics. However, the remaining patients were admitted with acute or chronic alcohol abuse, without de-compensated liver disease; and after initial management in the hospital, they were followed up in nurse-led clinics for rehabilitation after improvement in their alcohol-related disease and regular blood monitoring.

Septicemia is not only associated with high mortality[1] but it can also induce liver damage and liver injury by various mechanisms, including release of per-oxynitrite by leucocytes resulting in damage to the membrane of tissue cells,[3] apoptosis of the immune system[4] and tissue ischemia due to microvascular obstruction.[5,6] It is believed that microvascular obstruction is triggered by the release of pro-inflammatory mediators such as tumor necrosis factor (TNF) α and IL-1 by tissue macrophages in response to septicemia.[7,8]

The blockage of micro-circulation in response to bacterial infection may represent an adaptive phenomenon, which prevents the bacteria from entering into the systemic circulation; therefore, infection is localized to a limited area of the body.[12–15] Furthermore, there is evidence that if microvascular blockage in response to inflammatory response is widespread, then it can result in multi-organ failure and death.[16,17]

In our study, mortality rate was 31% in patients with bacterial sepsis, mostly in elderly patients with pneumonia. Furthermore, we also analyzed the effect of elevated liver biochemistry and bilirubin on mortality of these patients with sepsis. Transaminases did not have any effect on mortality. However, development of jaundice in elderly patients was associated with better prognosis. This result may represent robust immunity in these elderly patients with sepsis, which may be responsible for their survival. Similarly, it has been shown previously that because of potent immune response in some patients with acute hepatitis C, development of jaundice was associated with spontaneous clearance of virus.[18]

In all patients admitted to acute medical receiving unit, the cause of elevated LFT parameters was established, and after management these patients either returned back to normal or improvement was seen in most of the patients who survived. Various other conditions that resulted in elevated liver enzymes included congestive cardiac failure with liver congestion, hepatocellular carcinoma, liver metastasis from various primary tumors and drug overdoses. Furthermore, a few patients had elevated liver blood test parameters as a side effect of certain drugs such as azathioprine, statins and tegretal. Lastly, a few patients with non-alcohol fatty liver disease secondary to diabetes were admitted with acute complications of diabetes. In most of these patients except terminally ill patients, LFT parameters were either normalized or improved after treatment.

In conclusion, our study showed that patients with elevated LFT parameters admitted to our acute medical unit were being managed and followed up in accordance with AGA guidelines. Furthermore, jaundice may be associated with increased survival in elderly patients with bacterial sepsis. However, studies involving larger number of patients with bacterial sepsis are required to confirm this result.
